# Hemiplegic shoulder pain, a combined approach with suprascapular nerve block and intra-articular corticosteroid injections: a case series

**DOI:** 10.3389/fneur.2025.1620168

**Published:** 2025-06-25

**Authors:** Matthieu Gahier, Andy Lecler, Guillaume Gadbled, Paul Arnolfo, Raphael Gross

**Affiliations:** ^1^Department of Neurological Physical Medicine and Rehabilitation, Saint Jacques Hospital, University Hospital of Nantes, Nantes, France; ^2^Department of Neurological Physical Medicine and Rehabilitation, Côte d’Amour Rehabilitation Center, Saint-Nazaire, France; ^3^Department of Orthopedic Surgery, University Hospital of Nantes, Nantes, France; ^4^Department of Rheumatology, University Hospital of Nantes, Nantes, France; ^5^Laboratory Movement-Interactions-Performance (MIP), EA4334, University of Nantes, Nantes, France

**Keywords:** hemiplegia, stroke, traumatic brain injury, shoulder pain, nerve blocks, intra-articular injections

## Abstract

**Background:**

Hemiplegic shoulder pain (HSP) is a prevalent and disabling condition affecting patients after stroke or traumatic brain injury. There is currently no consensus regarding infiltrative strategies. A combined approach, involving suprascapular nerve block and intra-articular corticosteroid injection, has been proposed for HSP and capsulitis, yet evidence remains limited.

**Objective:**

This study presents the results (efficacy and safety) of this combined approach to alleviate pain and improve passive range of motion (PROM).

**Methods:**

A retrospective, multicenter observational study (36 patients).

**Results:**

At 1 month, the mean pain intensity (visual analogue scale VAS) significantly decreased from 6.5 ± 1.5 at baseline to 1.9 ± 2.1, and PROM showed significant improvement across all three planes (mean PROM gains: 28.4° in abduction, 29.2° in flexion, and 13.4° in external rotation). The benefits were largely maintained at 3 months, and no serious complications were observed (one vasovagal episode).

**Conclusion:**

The combined approach is a clinically feasible, safe, and effective method for treating HSP in PRM settings.

## 1 Introduction

Shoulder pain on the hemiplegic side is a common complication of brain injury, commonly referred to as hemiplegic shoulder pain (HSP). It affects up to 60% of patients with traumatic brain injury (TBI) and between 30 and 70% of patients with stroke (1, 2). HSP symptoms (pain and shoulder stiffness) impact patient’s autonomy in daily activities, their quality of life, and their rehabilitation outcomes (1–3).

HSP is a complex disorder, challenging to treat, with causes often multifactorial including both neurological and mechanical factors such as spasticity, rotator cuff injury, adhesive capsulitis, or complex regional pain syndrome (CRPS) (4). Among the various causes, adhesive capsulitis (frozen shoulder) has been identified in 43–77% of stroke survivors and is associated with more pronounced PROM limitations and a longer duration of HSP (4). Though Fitterer et al. (5) propose differentiating the components of HSP and treating them separately, there is currently no consensus on HSP treatment (6).

First-line treatments usually include physical therapy combined with analgesics, however this approach is often insufficient to treat the pain and the stiffening of the shoulder (2). In a study evaluating the characteristics of shoulder pain in 87 patients with TBI, Leung et al. (2) reported that two-thirds of patients presented with HSP upon admission (mean time from injury: 45 days, SD 24), and that pain decreased by only 1.2 points over the course of inpatient rehabilitation (mean length of stay: 34 days). Adey-Wakeling et al. (1) also reported, that among 148 patients with post-stroke HSP, nearly one third of patients had persistent shoulder pain 1 year after their stroke. In both studies, passive range of motion (PROM) in abduction and external rotation was shown to be correlated with the intensity of the pain felt by the patients (1, 2).

Second-line treatments include intra-articular injections and perineural injections. None has been proven to be superior to the other for the treatment of HSP (6). Treatments of interest include suprascapular nerve block (SSNB) and intra-articular corticosteroid injections (IAI). SSNB is known to reduce pain and improve PROM in various acute or chronic shoulder disorders (7–10). In HSP, a systematic review of eight randomized clinical trials showed that SSNB was effective in alleviating pain and increasing shoulder PROM (11). IAI has also been shown to be effective in patients with HSP in the subacute or chronic phase after stroke, leading to improved PROM and reduced pain, particularly for patients with tendinopathy or adhesive capsulitis (12, 13).

When compared to each other, neither SSNB or IAI has been proven to be superior to the other for the treatment of HSP (14–16). Two studies compared an approach combining SSNB and IAI to each technique alone but failed to demonstrate the superiority of the combined approach (15, 16). Recently, Shanahan et al. (17) used the combined SSNB and IAI approach for the treatment of adhesive capsulitis. Symptom duration was reduced by approximately 6 months compared to the control group, receiving no SSNB, with major improvements reported in pain, PROM, and functional scores (17).

After reviewing these positive results, we implemented in 2022 the same treatment protocol (i.e., combined SSNB and IAI approach) for the management of HSP in our physical medicine and rehabilitation (PMR) departments in Nantes and Saint-Nazaire (France). Two years after its implementation, we conducted a retrospective study to evaluate the effectiveness of this new protocol. The primary objective was to assess the effectiveness of the combined IAI and SSNB approach on shoulder pain at 1 and 3 months. Secondary objectives included assessing improvements in shoulder PROM and identifying predictive factors of treatment success.

## 2 Materials and methods

### 2.1 Study design

Data was manually abstracted (MG, AL, and RG) from the medical charts (both electronic and paper medical records) of patients treated for HSP between January 2022 and May 2024 in our two departments. Collected data included patients’ socio-characteristics, disease characteristics (e.g., delay of pain onset, Budapest criteria for CRPS…), and outcome evaluations. The diagnosis of CRPS was based on the Budapest criteria, requiring the presence of continuing pain disproportionate to any inciting event, and at least one symptom in three of the four categories (sensory, vasomotor, sudomotor/edema, and motor/trophic), as well as signs in at least two categories during clinical examination. Stroke severity was assessed using the NIH Stroke Scale (NIHSS), which provides a standardized quantitative measure of neurological impairment, with scores ranging from 0 (no deficit) to 42 (most severe). The following complications were investigated: infection, hematoma, and adverse events related to local anesthetics.

### 2.2 Patient selection

Eligible patients were adults (≥18 years) with motor impairment secondary to stroke or TBI and presenting with HSP, characterized by a pain score ≥ 4/10 (at rest, during mobilization or nursing) evaluated with a visual analogue scale (VAS) and limited shoulder PROM in at least two planes on the hemiplegic side. Patients could be in the subacute (15 days to 6 months) or chronic phase (after 6 months) post injury. They could be in- or out-patients. Exclusion criteria were recent shoulder fractures, shoulder surgery within 12 months, and local or general contraindications to articular injections, nerve blocks, or corticosteroids. In our clinical practice, no patients received NSAIDs or oral corticosteroids; only level 1 and 2 non-opioid analgesics were used, tailored to each patient’s individual pain profiles.

In both institutions, all treatment administrations and outcome assessments were performed by a senior physician (MG or RG).

### 2.3 Combined approach: description of the procedure

**Figure 1:**
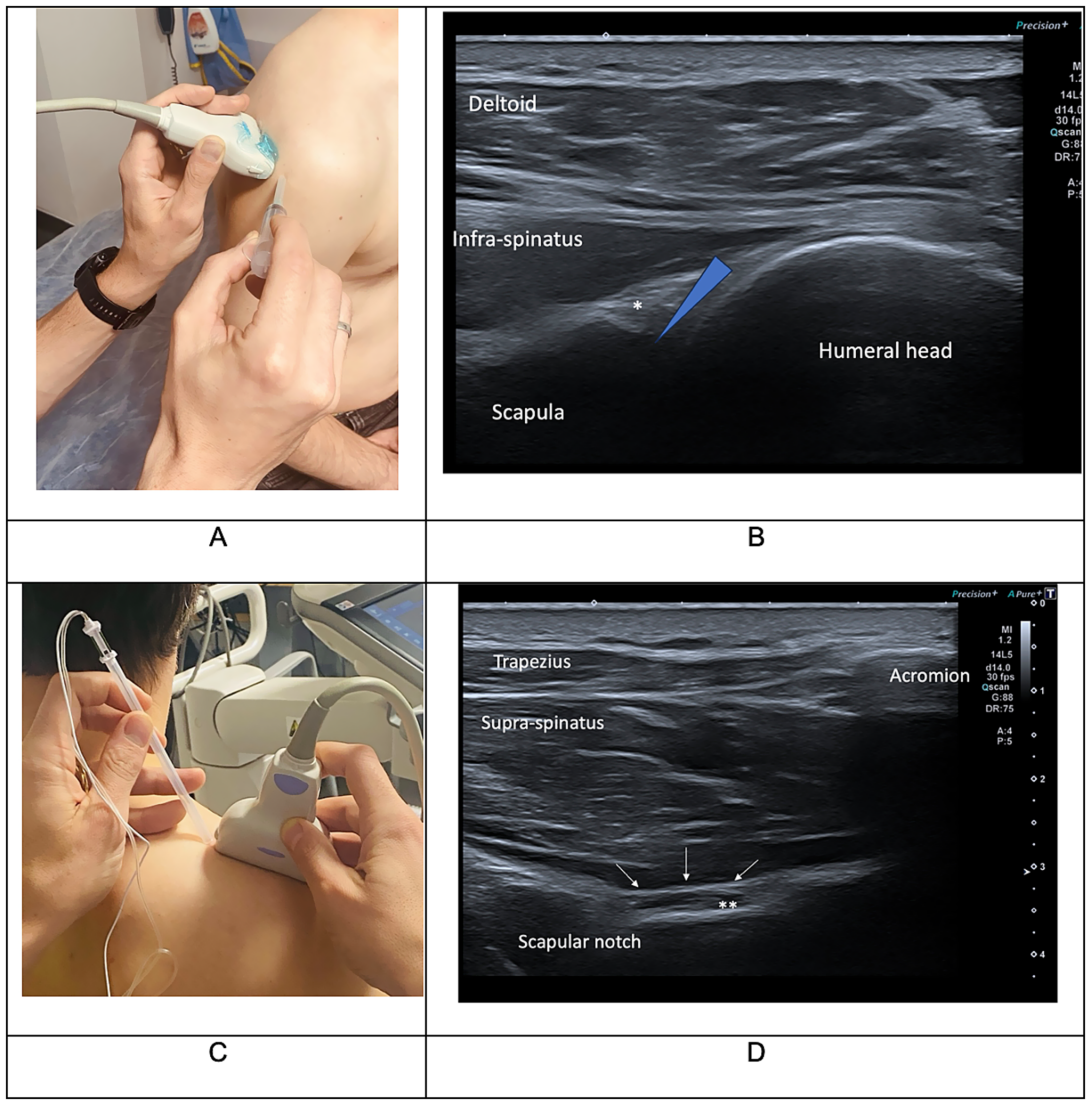
Clinical **(A,C)** and ultrasound **(B,D)** landmarks used for intra-articular corticosteroid injections (IAI) **(A,B)** and suprascapular nerve block (SSNB) procedure **(C,D)**. In picture **B**, the star indicates the glenohumeral labrum and the blue triangle indicates the targeted area for IAI. In picture **D**, the stars indicate the suprascapular nerve and the white arrows indicate the superior transverse scapular ligament on the suprascapular notch. Injections need to be performed in the suprascapular notch and followed by a ligament bulge. Photos courtesy of Dr. Etienne Savard.

The combined IAI and SSNB procedure consisted in an intra-articular injection of 2 mL lidocaine 1% and 7 mg of betamethasone followed by the SSNB performed at the suprascapular notch with 8 mL of lidocaine 1% and 7 mg of betamethasone. Injections were guided by ultrasound (Figure 1). These procedures were immediately followed by passive shoulder mobilization. Daily physical therapy focused on shoulder posture and passive mobilization was prescribed twice a day for the first 3 to 5 days. Thereafter, the patient resumed the previous multimodal rehabilitation program, adapted to their post-stroke stage and individual needs.

### 2.4 Outcome measures

Pain intensity and PROM were evaluated pre-procedure (baseline), at 1 h (H1), 1 month (M1), and 3 months (M3) post-procedure. Pain intensity was measured with a VAS ranging from 0 (“no pain at all”) to 10 (“unbearable pain”) and shoulder PROM was measured with a goniometer. Improvements were considered clinically significant if changes from baseline were >2 with the VAS and >15° with the PROM (cutoffs values were selected based on existing literature and clinical expertise). External rotation in RE1 was measured with the arm adducted and the elbow flexed at 90°. The 0° position corresponded to the forearm aligned with the trunk, negative values indicated inward rotation and positive values indicated outward rotation.

### 2.5 Data analysis

Data were summarized descriptively with means, standard deviations (SD), medians, and interquartile range (IQR) for continuous variables. Frequencies and percentages were reported for categorical variables. Changes from baseline at each follow-up time point were described and percentage of patients achieving clinically significant improvement was calculated. Statistical analyses were performed to compare pre-post treatment measurements using Wilcoxon tests for continuous variables and Fisher exact tests for categorical variables. Statistical analyses for comparison between multiple groups were performed using Kruskal–Wallis test. Factors associated with the success of the combined approach (PROM or pain at M1 or M3) were analyzed using a chi-squared test for categorical variables or a linear regression model for the continuous variable (spasticity score, associated botulinum toxin injection, age, pain duration and duration since lesion) with significance set at *p* < 0.05. Bonferroni corrections were applied to account for multiple comparisons. Due to the small sample size and non-normal data distribution, non-parametric tests were used instead of mixed-effects models.

### 2.6 Ethics

All individuals included in this study had been informed and accepted that their clinical data were used for research purposes. The study was conducted in strict accordance with the French law regarding non-interventional studies and data protection. Approval was granted by our local Ethics Committee on the 19th March of September 2024 (Number 24-36-03-191).

## 3 Results

### 3.1 Baseline characteristics of the study population

A total of 36 patients were included in the study. Mean age was 58.4 years ± 14.1 and 61% of patients were men. For 83% of patients, HSP occurred post-stroke (Table 1). Median time since-brain injury was 4 months (IQR: 3.0–6.3) and median pain duration was 3 months (IQR: 2.0–4.0). Mean pain intensity was 6.5 ± 1.5 on the VAS. Over half of the patients (58%) met the Budapest criteria.

**Table 1:** Population characteristics.

Population	Total *N* = 36
Demographics	
Men, *n* (%)	22 (61)
Age, mean (±SD)	58.4 (14.1)
Aetiology of hemiplegia, *n* (%)	
TBI	6 (17)
Stroke	30 (83)
Stroke severity, *n*/*N*^a^ (%)	
NIHSS <5	0/30 (0)
NIHSS 5–14	13/30 (43)
NIHSS 15–19	8/30 (27)
NIHSS >20	3/30 (10)
Hemiplegic side, *n* (%)	
Left	23 (64)
Right	13 (36)
Characteristics at baseline	
Time since brain injury, months, median (IQR)	4.0 (3.0–6.3)
Pain duration, months, median (IQR)	3.0 (2.0–4.0)
Positive Budapest criteria, yes, *n*/*N*^a^ (%)	19/33 (58)
Spasticity, yes, *n*/*N*^a^ (%)	19/31 (61)
Associated treatment with botulinum toxin, yes, *n*/*N*^a^ (%)	10/36 (28)
Significant paresis (abduction <3 on MRC scale) *n*/*N*^a^ (%)	17/33 (52)
X-ray findings, *n*/*N*^a^ (%)	
Normal	9/24 (38)
Gleno-humeral inferior subluxation	9/24 (38)
Degenerative injury	7/24 (29)

HSP, hemiplegic shoulder pain; NIHSS, National Institutes of Health Stroke Scale (assessed at patients admission in neurology department); TBI, traumatic brain injury. Spasticity were considered if present on pectoralis major, teres minor or subscapularis.

a *n*/*N*: number of patients/total number of patients with data available for analysis (modalities with missing data).

### 3.2 Primary outcome (pain reduction)

Mean VAS score decreased from 6.5 ± 1.5 at baseline to 0.5 ± 1.2 at H1 (*p* < 0.01), 1.9 ± 2.1 at M1 (*p* < 0.01), and 1.6 ± 2.7 at M3 (*p* = 0.03). The proportion of patients who reported a clinically relevant reduction in pain was 97% at H1, 97% at M1, and 85% at M3 (Table 2). No patient experienced an exacerbation of pain at H1 or at M1. Three patients however had recurrent pain at M3 and received a repeat injection.

**Table 2:** Pain and PROM evolution at baseline and during follow up.

Endpoint	Baseline	H1	M1	M3
Loss to follow-up	—	—	7	10
VAS^b^				
Overall score, mean (SD)	6.5 (1.5)	0.5 (1.2)	1.9 (2.1)	1.6 (2.7)
Clinically significant improvement, *n*/*N*^a^ (%)	—	34/35 (97)	28/29 (97)	22/26 (85)
Shoulder PROM^c^				
Flexion, mean (SD)	80.6 (25.0)	103.0 (27.3)	102.1 (34.7)	102.9 (32.9)
Flexion, mean gain (SD)		25.5 (25.2)	29.2 (24.9)	27.4 (31.8)
Clinically significant improvement, *n*/*N*^a^ (%)	—	10/19 (53)	14/19 (74)	13/21 (62)
Abduction, mean (SD)	65.2 (23.8)	87.5 (29.7)	86.6 (23.6)	89.5 (30.2)
Abduction, mean gain (SD)		26.3 (24.2)	28.4 (25.8)	31.0 (32.1)
Clinically significant improvement, *n*/*N*^a^ (%)	—	13/20 (65)	12/19 (63)	14/21 (67)
External rotation, mean (SD)	9.7 (20.7)	19.8 (20.6)	19.3 (22.1)	21.9 (27.1)
External rotation, mean gain (SD)		13.7 (13.8)	13.4 (13.3)	12.4 (20.1)
Clinically significant improvement, *n*/*N*^a^ (%)	—	10/23 (44)	11/22 (50)	12/21 (57)

PROM, passive range of motion; VAS, visual analogue scale.

a *n*/*N*: number of patients/total number of patients with data available for analysis (modalities with missing data).

b VAS ranging from 0 (“no pain at all”) to 10 (“unbearable pain”), >2 point decrease in VAS considered clinically significant. *p*-value for pre-post treatment comparisons was <0.01 at H1, M1, and M3.

c Measured by goniometer in degrees, >15° increase in PROM considered clinically significant.

### 3.3 Secondary outcome (PROM improvement)

Clinically significant improvements in shoulder PROM were observed across all planes of movement at all follow-up time points (Table 2). Mean PROM gains (SD) at M1 were 28.4° (25.8) in abduction, 29.2° (24.9) in flexion, and 13.4° (13.3) in external rotation (*p* < 0.01 for each comparison except for external rotation at M3 *p* = 0.04). These improvements were mostly maintained at M3, with clinically relevant PROM gains for 67% of patients in abduction, 62% in flexion and 57% in external rotation.

### 3.4 Safety

One patient experienced a vasovagal episode after the procedure, which resolved within a few minutes after being placed in the supine position.

### 3.5 Predictive factors of treatment efficacy

No predictive factors were significantly associated with better treatment outcomes.

### 3.6 Predictive value of immediate results

The result at H1 was predictive of the outcome for abduction at M1 (*p*-value <0.01, adjusted *R*-squared: 0.47) and M3 (*p*-value: 0.02, adjusted *R*-squared: 0.41) and for pain outcome (*p*-value = 0.02, adjusted *R*-squared: 0.27).

## 4 Discussion

Our findings suggest that the combined IAI and SSNB approach is effective for short- and medium-term pain relief and for improving shoulder PROM in patients with HSP. Benefits were observed up to 3 months post-treatment.

The significant pain reduction we report exceed those observed in previous studies evaluating the efficacy of SSNB or IAI alone. With the combined approach, in our study we observed a 4.6-point VAS reduction at 1 month. With SSNB alone, Terlemez et al. (18) and Adey-Wakeling et al. (19) reported a 2.9-point (pretreatment: 7.1, SD 1.8) and 3.7-point (pretreatment: 6.9, CI: 62.25–75.56) VAS reduction, respectively. With IAI alone, Lakse et al. (12) and Snels et al. (13) showed a 1.6-point (pretreatment: 5.2, SD 1.2) and 2.8-point (pretreatment: 5.1, IQR 4.2–6.3) VAS reduction, respectively. Our results were similar with previous studies assessing combined approach (15, 16).

Of note, our study population had a high NIHSS score (mean 12.9) which was not the case for the two studies previously described that failed to demonstrate the superiority of the combined approach compared to each technique alone (15, 16). Patients in both those studies had significantly less severe PROM limitations than our patients [for example baseline external rotation: 9.7° in our study vs. 41° (15) and 48° (16)], suggesting that their HSPs were less severe and probably not associated with CRPS or adhesive capsulitis (15, 16). The relative joint gain observed in our study—particularly in abduction and external rotation—may have a stronger clinical impact in patients with marked initial stiffness, as it contributes to pain reduction and improves comfort during daily care (1, 2). In severe cases, improved mobility helps maintain long-term relief by reducing pain triggers during activities such as dressing or washing.

While neither SSNB or IAI has proven superior to the other in managing HSP (14–16, 18), our results seem to indicate that combining these two approaches could achieve clinically relevant improvements in pain relief and PROM, compared to each technique alone (11–13). These results warrant confirmation through a randomized controlled interventional study.

The majority of our study population was in the subacute phase of brain injury (defined as <6 months post-lesion; *n* = 27/36), which calls for caution when generalizing these results to patients in the chronic phase (>6 months). Nevertheless, the time elapsed since the initial brain injury does not appear to be a determinant of treatment efficacy in our cohort. Notably, we observed clinically meaningful improvements in some chronic patients, including one case 14 years post-stroke. The management of HSP beyond one-year post-stroke has been rarely studied. Only Terlemez et al. (18) included patients slightly over 1 year after stroke (median between 13 and 15 months). Further research on this topic is warranted.

Our study did not identify any significant predictive factors for treatment success, suggesting that this combined treatment approach may be beneficial regardless of the patient’s characteristics.

Unlike Fitterer et al. (5), we support the hypothesis that the management of HSP should begin with pain relief, regardless of its characteristics, before addressing the issue of spasticity management, which may potentially be triggered by the pain itself.

The retrospective nature of this study and the lack of a control group are the main limitations of our study. Patients in the present study had severe HSP, as the mean VAS score at baseline was 6.5. PROM, particularly external rotation, can be assessed in various positions, which are not always clearly described by authors, thereby limiting the reliability of comparisons. Despite these limitations, pain relief and PROM gain obtained with this combined approach seemed to be superior to what we have observed in the past in our clinical experience with IAI alone, or with physical therapy associated with general medication.

## 5 Conclusion

In conclusion, our study supports the use of a combined IAI and SSNB approach, including lidocaine and betamethasone for both procedures for the management of HSP, with significant clinical improvements reported in both pain and shoulder PROM sustained up to 3 months in a majority of patients, regardless of the characteristics of HSP.

We recommend further research, in order to confirm these findings in larger, controlled and multicentered studies in order to refine the treatment strategy and evaluate the long-term benefits and the need for repeat injections.

## Data Availability

The raw data supporting the conclusions of this article will be made available by the authors, without undue reservation.
